# Unlocking the potential of CRISPR tools and databases for precision genome editing

**DOI:** 10.3389/fpls.2025.1563711

**Published:** 2025-07-21

**Authors:** Pooja Saraswat, Rajiv Ranjan

**Affiliations:** Department of Botany, Faculty of Science, Dayalbagh Educational Institute, Agra, Uttar Pradesh, India

**Keywords:** CRISPR/Cas, genome editing, computational tools, databases, guide RNA design, off target prediction

## Abstract

Recent breakthroughs in CRISPR/Cas genome editing have transformed molecular biology research and offer significant potential across biotechnology and medicine. This has created a broad spectrum of computational tools and databases that aim to optimize each phase of the genome-edited workflow, from guide RNA design and off-target prediction through screening analysis and biological validation. Here, we survey major CRISPR tools and analyse their features in the context of precision genome editing. CRISPOR and CHOPCHOP versatile platform that provides robust guide RNA design for several species, integrated off-target scoring, and intuitive genomic locus visualization. This review gives an overview of these new resources that have been developed, grouped based on their functionalities like design of guide RNA, off-target predictions, genome-wide screens, and visualizations of the data. Furthermore, we discuss new trends in database development like their integration with genome browsers and implementation of machine learning. This review thus gives a useful overview of the dynamic field of CRISPR/Cas genome editing tools. It also serves as a helpful guide for researchers looking to utilize these tools in their research.

## 1 Introduction

CRISPR-Cas is a versatile genome editing tool applicable to different species, including viruses, plants (Saraswat et al., 2024), and mammals (Mali et al., 2013). CRISPR revolutionizes farming by enabling scientists to change a plant’s DNA. It means we can grow crops that perform under hot conditions, drought, and pests, with improved yield, nutrition, and better longevity. Unlike traditional methods such as conventional breeding or GMOs, CRISPR uses the plant’s natural genes, making the technology faster, cleaner, and easier to approve. This technology isn’t merely a laboratory innovation instead, it’s a practical answer for enabling to produce more food sustainably for a growing global population (Gao, 2021). Also, CRISPR/Cas technology has come into importance as a breakthrough technology for cancer research due to its efficacy, flexibility, and specificity. CRISPR helps in cancer screening by making whole-genome functional analyses to map cancer-causing genes and pathways, diagnosis with ultrasensitive tools such as CRISPR-based biosensors and nucleic acid tests, and therapeutic intervention where genome editing corrects for errors in the genes or enhances immunotherapy, such as CAR-T cell engineering. Its ability to target specific genes with accuracy makes CRISPR a promising approach for individualized and targeted therapy for cancer (Yang and Zhang, 2023).

CRISPR uses an RNA-guided endonuclease, Cas9 and target sequences by complementary base pairing between a guide RNA (gRNA) and a 20-bp target sequence adjacent to protospacer adjacent motif (PAM) which is in the form of NGG (Wang and Li, 2021). The structure typically consists of direct repeats, ranging between 25 to 45 nt. These repeats are spaced by similar-length spacers that carry unique genomic material that is unique and most likely introduced via plasmids or viruses (Katyal et al., 2013). As a result, it’s crucial to consider both possible off-target sites and the gRNA sites in a gene when creating gRNAs for editing (Naeem et al., 2020). Based on the accessory cas genes and structure of the CRISPR-Cas locus, the CRISPR-Cas system is presently categorised into two main classes, which are further divided into six types and various subtypes (Koonin et al., 2017). However, new types may still need to be found (Makarova et al., 2020). Examining the CRISPR distribution among target strains is crucial from an evolutionary perspective. They are anticipated to be key players in prokaryotic adaptive immunity and could act as markers; therefore, having specialised identification methods and up-to-date databases is required.

The CRISPR-Cas system, consisting of direct repeats (DRs) and spacers, is crucial in determining the type of RNA molecules that can activate adaptive immunity (Couvin et al., 2018). A comprehensive investigation of DRs and spacers is essential. Software tools available online can be used to select specific CRISPR sites. Computational methods like CRT (Bland et al., 2007), CRISPRDetect (http://crispr.otago.ac.nz/CRISPRDetect/predict_crispr_array.html) (Biswas et al., 2016), MetaCRAST (Moller and Liang, 2017), and CRISPRdisco (http://github.com/crisprlab/CRISPRdisco) (Crawley et al., 2018) can predict prokaryotic genome CRISPR arrays. CRISPR-related databases like CRISPRdb http://crispr.upsud.fr/crispr (Grissa et al., 2007b), CRISPRI (Rousseau et al., 2009), and CRISPRCasdb (Pourcel et al., 2020) also integrate programs for CRISPR identification.

Also, to prevent viral infections, prokaryotes have developed antiviral defence mechanisms (Rostøl and Marraffini, 2019). The initial discovery of anti-CRISPR proteins was reported in phages and prophages associated with *Pseudomonas* (Bondy-Denomy et al., 2013). According to Samson et al. (2013), viruses have evolved anti-defence mechanisms, such as anti-CRISPRs, which prevent host CRISPR systems from functioning (Pawluk et al., 2018). Naturally occurring CRISPR-Cas inhibitors, or anti-CRISPRs (Acrs), may be used to create genome editing tools that are safer and easier to regulate.

The recent tools and databases to identify them are discussed later in the review.

## 2 Databases and tools for the prediction of CRISPR-Cas systems

**Figure 1:**
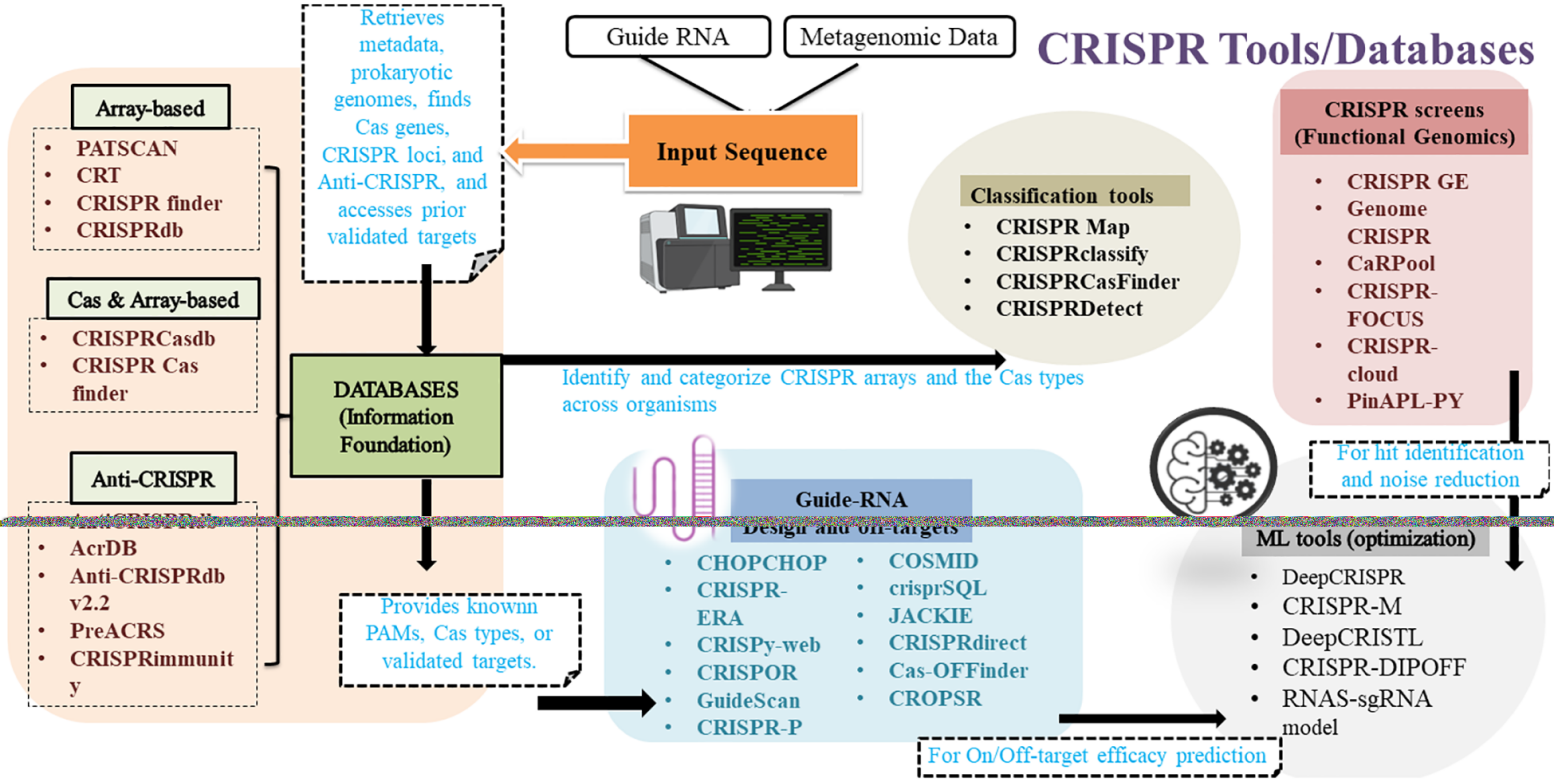
CRISPR system-related bioinformatics tools for different applications.

CRISPR-Cas systems play a vital role in the adaptive immunity of prokaryotes have been harnessed as a genome editing tool. By studying their natural functions, new CRISPR-based systems can be developed (Akram et al., 2023). To analyse these systems, it is essential to identify CRISPR arrays and their spacer sequences, which has led to various computational tools for recognising CRISPRs (**Figure 1**; **Table 1**). Several articles have provided a detailed overview of such tools in recent years (Alkhnbashi et al., 2020; Naeem and Alkhnbashi, 2023; Li et al., 2023). Early predictions of CRISPRs were made using tools such as PatScan (Godde and Bickerton, 2006), CRT (Bland et al., 2007), PILER-CR (Edgar, 2007), CRISPRFinder (Grissa et al., 2007a) and CRISPI (https://bio.tools/crispi) (Rousseau et al., 2009). Users can view all CRISPR identified in the genomes of bacteria and archaea using CRISPI. Microbial genomes are easily chosen using the accession number and the genome name. Following the selection of a genome, the findings are compiled into tables. Every CRISPR and its associated *Cas* genes are indicated. These are identified using specific Hidden Markov Model (HMM) profiles derived from the available genes. With the CRISPI tool, users can annotate their microbial sequences by identifying CRISPR repeats within the sequences they have submitted. The query sequence can be uploaded from a local machine or pasted into the input field; it must be in FASTA format (Rousseau et al., 2009). Previously developed bioinformatics tools like PatScan (Godde and Bickerton, 2006), CRT (Bland et al., 2007) and CRISPRFinder (Grissa et al., 2007a) often result in ambiguous CRISPR arrays, which are unable to identify the strand from which crRNA is derived. This is crucial for tasks like CRISPR conservation classification, detecting leader regions, identifying protospacers, and characterizing protospacer-adjacent motifs (PAM). CRISPRstrand is an advanced machine learning method designed to accurately predict the correct orientation of repeats within CRISPR loci, facilitating the identification of the strand from which mature crRNAs are produced. This adaptable technique effectively determines the transcribed strand of CRISPR loci making it a valuable tool for various tasks (Alkhnbashi et al., 2014). However, CRISPRstrand focuses more on classification and annotation, and not on the experimental design and analysis.

**Table 1:** List of bioinformatics tools and databases in CRISPR/Cas technique.

Sr. no.	Database	Type of organisms	Function	Speed	Limitations	Website link	Reference
1	CRISPRdb	Bacteria and Archaea	Identifies CRISPRs and spacers, a visualization tool	Moderate	Limited to CRISPR arrays; does not design guide RNA	http://crispr.upsud.fr/crispr	(Grissa et al., 2007b)
2	CRISPI	Bacteria and Archaea	A relational database to identify the CRISPR and CAS in personal sequences	NA	Limited to few organisms	https://bio.tools/crispi	(Rousseau et al., 2009)
3	CRISPR target	Bacteria, Archaea, Plants, Animals, Fungi, etc.	Predictions and analysis of crRNA targets	Moderate	not updated frequently	http://crispr.otago.ac.nz/CRISPRTarget/crispr_analysis.html	Biswas et al., 2013
4	COSMID	Human, mouse, *Caenorhabditis elegans*, and *Rhesus macaque*, etc.	Identify and Validate CRISPR/Cas Off-target Sites, allows custom genome input	Slow	limited experimental results	https://crispr.bme.gatech.edu/	Cradick et al., 2014
5	CRISPR-P	Plants	sgRNA designing tool	Fast	Plant-focused	http://cbi.hzau.edu.cn/crispr/	Lei et al., 2014
6	CRISPR multitargeter	Human, Oryza sativa japonica, *Gallus gallus*, *Mus musculus*, *Arabidopsis thaliana*	sgRNA designing tool from a set of similar sequences	Moderate	Limited batch processing	https://github.com/SergeyPry/CRISPR_MultiTargeter	Prykhozhij et al., 2015
7	WGE	Mouse and Humans	CRISPR sites in any genome, Visual guide design with genome browser integration	Fast	Limited to human and mouse genomes	https://wge.stemcell.sanger.ac.uk/	(Hodgkins et al., 2015)
8	CRISPRdirect	Any organism with a genome sequence	Design sgRNA with reduced off-target sites	Moderate	Basic scoring system	http://crispr.dbcls.jp/	(Naito et al., 2015)
9	CRISPRDetect		Detects CRISPR arrays and spacers	Fast	No off-target predictions	http://crispr.otago.ac.nz/CRISP RDetect/predict_crispr_array.html	(Biswas et al., 2016)
10	GenomeCRISPR	Mouse & Humans	Database for CRISPR/Cas9 screens	Fast	Limited species	https://genomecrispr.dkfz.de/#!/	Rauscher et al., 2016
11	CRISPy-web	Bacteria, Archaea	Design sgRNA for CRISPR/Cas systems	Fast	Limited species	https://crispy.secondarymetabolites.org/#/input	(Blin et al., 2016)
12	Cas Database	*Arabidopsis thaliana, Drosophila melanogaster*, human, mouse, etc.	gRNA library design tool for Cas9 nucleases	Fast	Focus on Cas systems only	http://www.rgenome.net/cas-database/	Park et al., 2016
13	caRpool	Human, Mouse	To analyse CRISPR/Cas9 screens	Moderate	Requires R knowledge	http://github.com/boutroslab/caRpools	(Winter et al., 2016).
14	CRISPR-FOCUS	Human & *Mus musculus*	Webserver for efficient screening experiments	Fast	Limited cell types	http://cistrome.org/crispr-focus/	Cao et al., 2017
15	CRISPRcloud	Human, Mouse	Reanalysis pooled CRISPR screens datasets	Moderate	Limited visualization	https://crispr.nrihub.org/	Jeong et al., 2017
16	CRISPOR	Human, Mouse, Rat, Fly, Worm, Zebrafish, Plants, etc.	Guide RNA selection for genome editing	Fast	Limited to fewer organism	http://crispor.tefor.net/	(Concordet and Haeussler, 2018)
17	CRISPRdisco	Bacteria, Archaea	Predicts CRISPR arrays	Moderate	Limited to fewer organism	http://github.com/crisprlab/CRISPRdisco	Crawley et al., 2018
18	Cpf1-Database	*Arabidopsis thaliana*, tomato, banana, human, mouse, zebrafish, etc.	Selecting guide RNA for CRISPR-Cpf1	Moderate	Cpf1-specific	http://www.rgenome.net/cpf1-database/	(Park and Bae, 2018)
19	PICKLES	Human, Mouse	Database for pooled CRISPR knockout libraries	Fast	Data-centric, not design-centric	http://pickles.hart-lab.org	Lenoir et al., 2018
20	Multiguidescan	Multiple organisms	Design guide RNA libraries from large genomes	Fast	Batch runs only	https://github.com/bioinfomaticsCSU/MultiGuideScan	Li et al., 2020
21	CRISPR Local	Human, Model organisms	Designing sgRNA in plants	Fast	More efficient for Batch runs	http://crispr.hzau.edu.cn/CRISPR-Local/	(Sun et al., 2019)
22	DrugThatGene	Human	Identification of small molecules, pathways and protein complexes from CRISPR screens	Moderate	Niche use-case	https://github.com/pinellolab/DrugThatGene	Canver et al., 2019
22	CRISPRCasdb	Bacteria, Archaea	Provide access to CRISPR and Cas genes	Fast	Cas-centric	https://crisprcas.i2bc.paris-saclay.fr/	(Pourcel et al., 2020)
23	AcaFinder	Bacteria, Archaea	For anti-CRISPR associated genes	Moderate	Acas that do not have Acrs in proximity, miss out, novel Aca are found based on the similarity to known sequence	https://github.com/boweny920/AcaFinder	(Yang et al., 2022)
24	AcrFinder	Bacteria, Archaea	Acr-Aca (Acr-associated regulator) operon prediction program	Moderate	relies on Aca references, requires that genomes have complete CRISPR-Cas systems	http://bcb.unl.edu/AcrFinder	(Yi et al., 2020)
25	PINCER	Bacteria, Archaea	CRISPR screening using efficient cleavage at protein residues	Moderate	Limited to human and mouse	https://github.com/veeneman/PINCER	(Veeneman et al., 2020)
26	AsCRISPR	Human	Allele-specific sgRNA designing	Fast	Focused on allele-specific targets	https://bio.tools/AsCRISPR	(Zhao et al., 2020).
27	CrisPAM	Bacteria, Archaea	SNP-derived PAM analysis tool	Fast	SNP-Derived PAM only	https://github.com/RoyRabinowitz/CrisPam	(Rabinowitz et al., 2020)
28	CRISPRclassify	Bacteria, Archaea	Repeat-based classification of CRISPR systems	Moderate	Uses BLAST and HMM alignments which miss out a substantial proportion of CRISPR loci in metagenomes which remain unclassified	https://github.com/CRISPRlab/CRISPRclassify	(Nethery et al., 2021)
29	AcrDB	Bacteria, Archaea, Virus	Anti-CRISPR operons in prokaryotes and viruses	Moderate	It misses Acr proteins that do not need Aca regulators, fail to identify anti-CRISPRs in genomes with incomplete or without CRISPR–Cas systems	http://bcb.unl.edu/AcrDB	(Huang et al., 2021)
30	dbGuide	Human, Mouse	A database of functionally validated gRNA sequences	Fast	Restricted to few organisms,	https://sgrnascorer.cancer.gov/dbguide	(Gooden et al., 2021)
31	CRISPRloci	Bacteria, Archaea	Comprehensive annotation of CRISPR-system	Moderate	Limited input size	https://rna.informatik.un-freiburg.de/CRISPRloci/	(Alkhnbashi et al., 2021)
32	CrisprVi	Prokaryote genome	Visualize and analyse CRISPR sequences	Moderate	Visualization only	https://sourceforge.net/projects/crisprvi/	(Sun et al., 2022)
33	SynBioStrainFinder	Synthetic biology strains (Bacteria, Yeast)	microbial strain database with information related to strain CRISPR/Cas system	Fast	Synthetic strains only	http://design.rxnfinder.org/biosynstrain/	(Cai et al., 2022)
34	CROPSR	Plants	Genome-wide sgRNA design and validation tool	Moderate	Issues with the long compute times	https://github.com/cabbi-bio/CROPSR	(Müller Paul et al., 2022)
35	JACKIE	Genome file	Evaluates off-target sites and their numbers, strong batch processing capabilities.	Moderate	Bulk-focused, not ideal for small-scale or simple edits.	https://github.com/albertwcheng/JACKIE2	Zhu and Cheng, 2022
36	PreAcrs	Genome and Metagenome projects	Identifies antiCRISPR proteins	Moderate	does not provide a visual and user-friendly website, one algorithm	https://github.com/Lyn-666/anti_CRISPR	Zhu and Cheng, 2022
37	CRISPRoffT	Human & Mouse	Comprehensive database for off-targets	Moderate	Not a prediction tool	https://ccsm.uth.edu/CRISPRoffT/	Wang et al., 2025
38	CRISPR-P 2.0	Plants	sgRNA design	Fast	Plant-focused	http://cbi.hzau.edu.cn/CRISPR2/.	Liu et al., 2017
39	DeepCRISPR	On-/Off-target (Cas9)	deep-learning model to predict off target detection	Moderate	Limited interpretability, mainly substitution mismatch handling	http://www.deepcrispr.net/	Chuai et al., 2018
40	CRISPR-M	Off-target (Cas9 with indels)	Predicts off-target for target sites with mismatches and indels	Moderate	Requires large memory; AlphaFold integration is time-consuming	https://github.com/lyotvincent/CRISPR-M	Sun et al., 2024
41	AcrNET	Anti-CRISPR protein	anti-CRISPR (Acr) proteins protein prediction, predicts their specific types	Moderate	Limited to short sequences; ≤3 per file	https://proj.cse.cuhk.edu.hk/aihlab/AcrNET/),	Li et al., 2023
42	DeepCRISTL	On-target	predict on-target efficiency for CRISPR-Cas9 editing using high-throughput datasets	Moderate-Fast	Requires well-correlated source-target datasets for TL	https://github.com/OrensteinLab/DeepCRISTL	Zhu et al., 2024
43	RNAS-sgRNA	On-target (Cas9)	NAS and RNN integrated model to provide a reliable method to predict on-targets	Fast	Moderate interpretability	https://github.com/shehlarafiq5/RNAS-sgRNA	Rafiq and Assad, 2025
44	CRISPR-DIPOFF	Small molecule inhibitor profiling	Deep learning model, that precisely predicts CRISPR-Cas9 off-target sites	Slow (lab-based)	Requires sequencing and *in vitro* digestion; not for direct prediction	(https://github.com/tzpranto/CRISPR-DIPOFF	Toufikuzzaman et al., 2024)

### 2.1 CRISPRDetect

CRISPRDetect is a web-based tool that automatically detects, predicts, and refines CRISPR arrays in genomes. This enables precise detection of CRISPR arrays, their orientation, repeat-spacer boundaries, and any substitutions, insertions, or deletions. Additionally, it provides a list of annotated cas genes. The tool is compatible with other analysis tools and can be utilized for target prediction. In a comparison of 3870 “good” arrays with predictions from other programs, such as PILER-CR and CRT, all programs identified 1782 common arrays. CRISPRDetect demonstrated the highest agreement with PILER-CR and CRT, identifying 1407 additional arrays in common and 345 arrays more than other methods. CRISPRDetect primarily focuses on CRISPR arrays with less information about Cas proteins and could limit the classification of more diverse subtypes (Biswas et al., 2016). Unlike CRISPRDetect and CRISPI, which focus only on identifying CRISPR arrays, CRISPRminer and CRISPRBank (Zhang et al., 2018) use various programs to identify both CRISPR and Cas. These websites have different interfaces and tools, with CRISPRBank containing CRISPR cas genes and arrays from 2733 strains, while CRISPRminer provides a database that contains CRISPR and cas genes sourced from prokaryote genomes and classifies these systems into six types and identifies self-targeting regions (Zhang et al., 2018). The CRISPR identification tool, CRISPRidentify (https://github.com/BackofenLab/CRISPRidentify), employs machine learning to identify and distinguish genuine CRISPR arrays from false ones in the genomic sequences with higher specificity. It uses various machine learning approaches such as Support Vector Machine, K-nearest Neighbours, Naive Bayes, Decision Tree, Fully Connected Neural Network, Random Forest, and Extra Trees classifiers to accurately investigate and distinguish true CRISPR arrays from false positives. This data-driven approach significantly enhances the precision and reliability of CRISPR array identification. This process involves three main stages: detection, extraction, and classification, using carefully curated datasets of confirmed CRISPR arrays as well as non-CRISPR sequences. The user receives a report with the detected CRISPR arrays together with the annotation. The tool exhibits a significantly lower false positive rate compared to other methods, as it accurately identifies candidates for CRISPR arrays that haven’t been found by other tools in addition to those that have already been found (Mitrofanov et al., 2021). CRISPRidentify is capable of addressing common issues encountered by previously existing tools, including the existence of identical spacers inside the array for CRISPR array identification. CRISPRidentify distinguishes CRISPR arrays by focusing on arrays with a few repeated spacers, unlike other tools that do not assess spacer similarity. This makes CRISPRidentify more effective than CRT and CRISPRCasFinder, which do not perform this kind of evaluation (Mitrofanov et al., 2021).

### 2.2 CRISPRdb and CRISPRCasdb

The CRISPRdb database (http://crispr.upsud.fr/crispr) is a monthly database that is updated using freshly released genome sequences. The software offers various tools to create a library of CRISPR arrays, align flanking sequences, search for similarities and analyse the structural organization (Grissa et al., 2007b). A new database called CRISPRCasdb makes both CRISPRs and Cas genes accessible. It is a feature of the CRISPR-Cas++ website, where users can download programs to analyse sequences that have been submitted. The database processes public whole genome assemblies using CRISPRCasFinder, a tool that finds cas genes and CRISPR arrays. Data recovery and BLAST searches against lists of spacers and repeats are accomplished by CRISPRCasFinder. The strains are arranged either taxonomically or alphabetically. 36,605 full prokaryote genomes, comprising 36,052 bacteria and 553 archaea, are available in CRISPRCasdb. The application includes two primary programs: CRISPRCasFinder and Database Tools. CRISPRCasFinder identifies CRISPRs and cas genes within genomic sequences, whereas Database Tools retrieves metadata, prokaryotic genomes, and taxonomy from the NCBI site. Additionally, the application supports BLAST searches of direct repeats and spacers available in the database. CRISPRCasdb presents analysed genomes in alphabetical lists or taxonomic classifications, allowing users to discover interesting CRISPR-Cas systems. Filters on CRISPR arrays and Cas presence/absence allow for relevant information selection (Pourcel et al., 2020). CRISPRCasdb also provides more comprehensive information of types and sub-types in comparison to CRISPRdb.

### 2.3 CRISPRloci

The CRISPRloci (https://rna.informatik.un-) server represents a significant advancement in CRISPR locus prediction, using a sophisticated Machine Learning technique. It accurately predicts and evaluates all potential CRISPR loci, offering precise assessments of CRISPR array orientation, definition of CRISPR leaders, and cas genes annotation. This tool provides comprehensive information about the CRISPR array, including Cas subtypes, repeat structure, orientation, virus-host interactions and self-targets. To enhance accuracy, CRISPRloci generates multiple candidates for each region of the genome and employs a data-driven approach to eliminate incorrect identifications, resulting in robust filtering of inaccurate candidates while maintaining sensitivity and specificity. CRISPRloci is an efficient tool aimed at enhancing the identification and representation of CRISPR arrays within genomic DNA. Notably, CRISPRloci can autonomously manage the complete deletion of spacers regardless of their position and can identify truncated repeat sequences. Leveraging the Cas boundary tool for analysing protein sequence input for CRISPR cassette boundaries, CRISPRloci employs CRISPRcasIdentifier (Padilha et al., 2020) to classify and predict potentially missing proteins. Moreover, it simulates potential virus-host interaction by the identification of protospacer regions within provided viral sequences, helping in studying of evolutionary aspects of viral targets (Alkhnbashi et al., 2021). Along with the support of a wide range of organisms for comparative analysis, it lacks functional analysis. The tool shows a significant improvement in detecting CRISPR-Cas interference modules when compared with CRISPRCasFinder. In particular, it improved the detection of single-module cassettes by 16% and the identification of multiple interference modules by more than 60%. The tool’s enhanced sensitivity and accuracy are demonstrated by this notable improvement, especially when it comes to detecting intricate CRISPR-Cas architectures that are frequently overlooked by traditional detection pipelines.

### 2.4 CrisprVi

Two computational tools, CRISPRviz (Nethery and Barrangou, 2019) and CRISPRStudio (Dion et al., 2018), offer interactive analysis capabilities for CRISPR sequences. CRISPRviz enables prediction, visualization, and manipulation of CRISPR sequences, utilizing MinCED which extracts CRISPR direct repeats and spacers and facilitates visual comparison of sequence graphics. However, it may generate confusing colour and symbol combinations for complex scenarios, and its reliance on MinCED for the detection of CRISPR impacts visualization accuracy. In contrast, CRISPRStudio solely presents spacers graphically and does not offer sufficient functionalities (customizing visualization colours CROPSR) for users to manipulate and analyse DRs/spacers. CrisprVi (https://sourceforge.net/projects/crisprvi/) is a Python package designed to visualize CRISPR direct repeats and spacers, offering features such as genomic order, IDs, and coordinates. It includes components like a GUI for visualization, a command parser, and local databases for storage. Unlike other tools such as CRISPRviz and CRISPRStudio, CrisprVi emphasizes interactivity and provides basic statistics on CRISPR and consensus sequences from input strains. This user-friendly tool supports researchers in exploring and analysing CRISPR sequences, facilitating the study of novel CRISPR-Cas systems in prokaryotes (Sun et al., 2022).

## 3 Database for AntiCRISPR proteins

### 3.1 Anti-CRISPRdb and Anti-CRISPRdb v2.2

Prokaryotes have an antiviral system called CRISPR-Cas that is extensively used for genome editing. Anti-CRISPR proteins are used to regulate Cas nuclease activity in CRISPR-Cas genome editing, ensuring safer and more controlled editing processes. These proteins, found in prophages can inhibit the CRISPR-Cas systems of their hosts. They were first identified by Bondy-Denomy et al. (2013), demonstrating anti-I-F activity in a *Pseudomonas aeruginosa* phage, marking a significant discovery in the field. Anti-CRISPR proteins block CRISPR-Cas systems, potentially enhancing gene editing precision. A comprehensive collection of these proteins is available in the anti-CRISPRdb, an online database facilitating easy access to protein sequences, coding regions, source organisms, taxonomy, and more. Users can browse, download, and upload data related to anti-CRISPR proteins through its user-friendly interface, enabling efficient research and application (http://guolab.whu.edu.cn/anti-CRISPRdb/) (Dong et al., 2018).

More details on mechanisms, inhibitory stages, the inhibitory ability for Acr-Cas/Acr-CRISPR systems, and the Acr neighbour estimate are available in the updated version, Anti-CRISPRdb v2.2. More entries and families are included, both from recent literature as well as via homologous alignment. Anti-CRISPRdb v2.2 incorporates the prediction of Acr neighbours, enabling users to identify new Acrs from these candidates. To motivate the advancement of prediction tools, the revised database now contains experimental data on the inhibitory strength and stage for Acr-Cas/Acr-CRISPR (Dong et al., 2022).

### 3.2 AcrDB

Researchers developed the online database AcrDB (http://bcb.unl.edu/AcrDB) by using AcrFinder (http://bcb.unl.edu/AcrFinder), a novel software for predicting Acr-Aca (Acr-associated regulator) operons (Huang et al., 2021). They analysed more than 19,000 genomes from prokaryotes and viruses for this purpose. The database depends on CRISPR-based self-targeting techniques and homology search. AcrDB is a comprehensive database featuring 39,799 Acr-Aca operons containing Aca or Acr homologs, making it the largest collection of its kind. The database offers a user-friendly web interface equipped with various options to browse, search and download. Unlike focusing solely on individual Acr protein families, AcrDB emphasizes the genomic context of Acr and Aca candidates. It integrates data from three independent programs, each employing unique data mining algorithms for robust validation. AcrDB covers computationally predicted Acr-Aca operons across more than 7,000 RefSeq genomes of prokaryotes and their viruses (Huang et al., 2021). AcrDB being a specialized database, limits itself to providing experimental data and tools for functional validation of the Acr proteins. Also, it fails to find Acr for organisms other than prokaryotes with incomplete CRISPR/Cas systems (Yin et al., 2019).

The discovery of Acrs can be sped up by using machine learning to recognize the new Acrs from protein sequences. PreAcrs (https://github.com/Lyn-666/anti_CRISPR) is a unique machine-learning predictor that can directly detect anti-CRISPR proteins from provided protein sequences. PreAcrs considerably predict accurately anti-CRISPR proteins and surpass other previous approaches (Zhu et al., 2022). It provides annotation of Acr proteins specifying their targets and mechanism of inhibition and offers better visualization to show their interaction with CRISPR systems. Experimental validation lacks which may limit the tool’s efficiency in some cases. A new web-based server was created for type II CRISPR-Cas discovery, Acr prediction, and the analysis of significant CRISPR-related molecular processes. CRISPRimmunity (http://www.microbiome-bigdata.com/CRISPRimmunity/index/) offers a thorough co-evolutionary view of the CRISPR-Cas and anti-CRISPR systems. Experimental validation of the cleavage activity of several recently discovered class 2 CRISPR-Cas loci utilizing CRISPRimmunity has been conducted *in vitro* (Zhou et al., 2023). CRISPRimmunity makes it simple to use for further data mining and experimental design by providing catalogues of pre-identified CRISPR systems that can be browsed, resources or databases that can be downloaded, an in-depth tutorial, graphical interface, and results that can be exported and accessed in machine-readable formats (Zhou et al., 2023) which were lacking in other tools like AcrDB (Huang et al., 2021) and PreAcrs (Zhu et al., 2022).

AcrNET (https://proj.cse.cuhk.edu.hk/aihlab/AcrNET/), introduced as a deep learning framework for predicting anti-CRISPR (Acr) proteins, mitigates significant shortcomings of previous methodologies by utilizing transformer learning algorithm to solve data scarcity and enhance generalizability. AcrNET surpasses earlier models restricted by minimal datasets and inadequate predictive accuracy by proficiently classifying input protein sequences as Acrs and predicting their specific types. AcrNET enhances predictive performance and minimizes dependence on lengthy biological validation by integrating structural information via transformer-based algorithm on huge protein databases, thereby generating useful sequence representations. The workflow is enhanced by integrating techniques such as AlphaFold and protein–protein docking simulations to predict Acr–CRISPR-Cas interactions, yielding significant insights including docking locations and energies before experimental validation. Despite existing computational limitations on input size and the resource-intensive nature of AlphaFold, the study underscores the promise of integrating deep learning with structural bioinformatics to expedite Acr discovery and reduce off-target effects in genome editing (Li et al., 2023).

## 4 Databases for CRISPR screens

CRISPR/Cas9 system has emerged as an efficient technique for genetic screening in mice, humans, and zebrafish, among other organisms. The rapid development of CRISPR/Cas9-derived functional data is based on its accessibility. Resources like CrisprGE (http://crdd.osdd.net/servers/crisprge/) (Kaur et al., 2015) and WGE (https://wge.stemcell.sanger.ac.uk/) (Hodgkins et al., 2015) have been developed to understand and design CRISPR experiments. Nevertheless, there hasn’t been a database that compares screening outcomes throughout the entire genome. A database called GenomeCRISPR (https://genomecrispr.dkfz.de/#!/) is used for CRISPR/Cas9 high-throughput screening studies. It includes information on the 700,000-single guide RNAs that were utilized in approximately 500 research studies conducted in 421 distinct human cell lines. The search for genes or genomic regions is among the data mining techniques offered by GenomeCRISPR. Users can compare the outcomes of several screens or the effects of various sgRNAs on the target gene by using phenotypic and genome views. However, it is restricted to human cell lines only. CaRpools (http://github.com/boutroslab/caRpools), an R package tailored for CRISPR/Cas9 screens, enables intuitive exploration and analysis of pooled screening data. It includes features like biological insights, links to external databases, detailed screening-related information, and reports. CaRpools supports customization with new hit-calling methods and efficient sgRNA designs. Its transparent analysis reports aid in creating databases for CRISPR/Cas9 screens and simplify dataset meta-analyses (Winter et al., 2016). caRpool is user-friendly for both beginners and experts, including the comprehensive screening and can be extended to develop new algorithms for hits and export efficient sgRNA designs to other databases.

CRISPR screens based on the CRISPR/Cas system enables efficient and cost-effective genome-wide gene function analysis. A web-based tool called CRISPR-FOCUS (http://cistrome.org/crispr-focus/) discovers single-guide RNAs (sgRNAs) based on efficiency, conserved sequence specificity, genome variations, and SNP for use in CRISPR screen experiments (Shukla et al., 2022). In addition, CRISPR-FOCUS offers additional essential sequences in the construct together with pre-defined positive and negative control sgRNAs. The features allow users to directly synthesize gRNA according to CRISPR-FOCUS output. It can design up to 30 sgRNAs for each 1000 target genes and takes about twenty seconds. CRISPR-FOCUS offers a high throughput method for designing sgRNA libraries, allowing users to effectively carry out targeted screen experiments aimed at different genes (Cao et al., 2017). CRISPRcloud (https://crispr.nrihub.org/) is designed for analysing pooled screening data, processing raw next-generation sequencing files, and presenting results through a secure web platform. It supports the extraction, clustering, and analysis of data from pooled CRISPR screening experiments, enabling quick reanalysis of datasets (Jeong et al., 2017). Later, PinAPL-Py (http://pinapl-py.ucsd.edu) was developed with improvement in terms of automatic extraction of sgRNA, flexibility and customization as per experimental needs, comprehensive workflow and sequence quality control (Spahn et al., 2017). PinAPL-Py can be preferred over the CRISPR cloud when working with large datasets. CRISPR/Cas9 functional genomic screens are essential for discovering drug targets, but their sensitivity can be limited by guide RNAs that fail to effectively disrupt gene function. A recent study reanalysed CRISPR tiling data using a comprehensive feature database to identify optimal guides and targets for predicting activity. These findings were integrated into a unified guide design algorithm to enhance the sensitivity of genome-wide CRISPR libraries. This led to the development of the ProteINConsERvation (PINCER) (https://github.com/veeneman/PINCER) genome-wide CRISPR library, which optimizes enzymatic efficiency while targeting conserved protein regions. By leveraging evolutionary conservation, PINCER improves protein hit identification, reduces false positives, and enables the discovery of high-confidence hits. Findings indicate that PINCER outperforms other genome-wide CRISPR libraries in effectiveness (Veeneman et al., 2020).

## 5 Tools for classification of CRISPR systems

CRISPRmap is a tool designed to analyse the structure and sequence conservation of CRISPRs using an extensive dataset of repeat sequences. It identifies key features of CRISPR-Cas systems, including, RNA structure motifs, and cleavage sites and relates Cas subtypes and evolution of CRISPR. The comprehensive overview by CRISPRmap allows for inferences about CRISPRs within the same sequence families and helps to identify potentially novel and highly divergent CRISPR-Cas systems (Lange et al., 2013). Detecting and classifying CRISPR-Cas systems in metagenomic data is essential for understanding their various genome editing applications, but for that computational issues still remain. A main problem is the complex and variable nature of metagenomic data, as the sequence data is from various unidentified organisms with different genomic structure. This makes the identification of CRISPR arrays and associated Cas genes a challenge as reference genomes are not available for identification (Nielsen et al., 2014). Also, the short contigs, a result of fragmented assemblies within metagenomics, prevent recovery of all CRISPR arrays or Cas operons which limits further evolutionary studies (Lam and Ye, 2019). Another problem is false positives due to tandem repeats present within microbial genomes and often increase chances of misunderstood as CRISPR arrays, especially when working with poor quality sequence datasets (Zhang and Ye, 2017). Overcoming these hurdles is likely to involve integrating better assembly approaches with better CRISPR identification algorithms and machine learning frameworks that are resistant to noisy, and taxonomically heterogeneous datasets.

Traditional approaches typically depend on identifying adjacent Cas genes to detect CRISPR loci and rely on BLAST and HMM alignments, which often leave many CRISPR loci in metagenomes unclassified (Nethery et al., 2021). But a new machine learning approach called CRISPR classify (https://github.com/CRISPRlab/CRISPRclassify) was created that solely uses repeat sequences to find and group CRISPR loci, without utilizing Cas genes. This method finds unclassified loci that other methods miss and shows important properties of CRISPR repeats that can be used to classify subtypes. CRISPR classify uses a one-*vs*-all (OVA) binarization approach, where for each subtype of CRISPR-Cas, an independent XGBoost classifier is trained based on binary classifications. This facilitates learning and better discrimination for subtypes, particularly for complex or borderline cases (Mitrofanov et al., 2021). The CRISPR classify pipeline includes three steps: identifying CRISPR arrays, extracting features, and classifying with a stratified model (Nethery et al., 2021). One key strength of CRISPR classify is that it classifies systems using just repeat sequences, thereby eliminating any need for dependence on Cas gene annotations. The repeat-based classification XGBoost outperformed all other nonlinear models and deep learning models and had strong generalizability, such that performance stayed even for repeat sequences that were largely divergent from training data. This is good for the potential for discovery of new occurrences of CRISPR loci in uncharacterized or in metagenomic data.

## 6 Guide-RNA design and off-target identification tools

CRISPR/Cas9-based genome editing has emerged as a significant milestone in the molecular field, enabling precise modifications to diverse genomes (Saraswat and Ranjan, 2022). Initially evolved in prokaryotes as a defence mechanism against bacteriophage infections, this system has found extensive use in the workflows of genome engineering. The spCas9 endonuclease from *Streptococcus pyogenes* is particularly prevalent in these applications. To employ Cas9 effectively, efficient single-guide RNAs (sgRNAs) need to be designed for the target gene. Importantly, for this information about PAM sequence is also required.

The sequence length of the PAM motif varies among different Cas protein variants, with distinct recognition sites. For example, the widely used SpCas9 (*Streptococcus pyogenes*) recognizes the 3 bp NGG PAM, while SaCas9 (*Staphylococcus aureus*) requires the longer NNGRRT sequence. Other Cas9 orthologs, such as NmCas9 and StCas9 recognizes NNNNGATT and NNAGAAW, respectively. In contrast, Cas12a (also known as Cpf1) and other type V effectors recognize T-rich PAMs (e.g., TTTV) while type VI Cas13, which targets RNA and not DNA, does not require a PAM at all. This diversity in PAM recognition sites has functional role in genome editing applications as the shorter PAMs (SpCas9 like SpCas9-NG or SpRY) covers broader genome. At the same time, precise PAM requirements can enhance targeting specificity and reduce off-target effects. PAM variability is also reflective of host genome adaptation in naturally occurring CRISPR-Cas systems. Thus, understanding and exploiting PAM sequence diversity is fundamental to optimizing CRISPR-based tools for research. For PAM identification, CrisPam (https://github.com/RoyRabinowitz/CrisPam), a computational tool, has been developed, which facilitates allele-specific targeting using CRISPR-Cas systems. Researchers who want to focus on PAM sequences related to the recognition of CRISPR systems can find CrisPAM a valuable tool. The tool scans sequences to detect multiple PAMs generated by both reference and variant sequences. Successful targeting occurs when at least one PAM is created by the variant nucleotide, ensuring specific binding of the Cas enzyme to the variant allele. CrisPam streamlines the design of guide RNAs for precise targeting of the allele and explores a diverse array of unique PAMs from different Cas enzymes (Rabinowitz et al., 2020).

In addition, an efficient gRNA synthesis requires fewer off-targets for which several tools have been developed. The Cas-OFFinder is a tool designed to detect potential off-target sites for Cas9 RNA-guided endonucleases. It can be accessed for free either as a command-line program or via a website. This tool allows searches in any sequenced genome without restrictions on PAM sequences or the number of mismatches. Cas-OFFinder enhances genome editing precision by addressing off-target mutation concerns. Unlike other tools such as TagScan (Cradick et al., 2011), Bowtie (Langmead, 2009), and CUSHAW (Liu et al., 2012), it does not limit the number of mismatches in its searches. It also considers the variability in PAM recognition by different Cas9 proteins for a more thorough off-target site search. It applies to a wide range of organisms, ensuring quick and comprehensive identification of potential off-target sites (Bae et al., 2014). Another tool to address the concern of off-target cleavage is COSMID (CRISPR Off-target Sites with Mismatches, Insertions, and Deletions), available at http://crispr.bme.gatech.edu. Based on the user-provided guide strand and specified parameters, COSMID scans genomes to identify potential off-target sites with the designated number of mismatched bases and insertions or deletions compared to the guide strand. What sets COSMID apart is its exhaustive genomic search for off-target sites due to changes in base pair (mismatches, deletions, and insertions), and also provides primers for later experimental work. TagScan algorithm is used by COSMID which helps in minimizing run times when performing for exhaustive genome searches. The run times without primer design off takes averaged 4 seconds (Cradick et al., 2014).

CHOPCHOP, simplifies the selection and design of optimal TALEN and CRISPR/Cas9 target sequences across various organisms. It accepts diverse inputs, predicts off-target effects, and offers interactive visualization and primer design to streamline genome engineering (Montague et al., 2014). CHOPCHOP can process multiple gene inputs, provides a dynamic graphical display aiding in the easy selection of optimal target sites, and is particularly useful for designing two sgRNAs. The tool automatically generates primers and visualizes restriction sites, therefore simplifying the genome engineering process. CHOPCHOP v2 brings significant enhancements to improve target accuracy and efficiency. It supports various CRISPR effectors and allows custom-length sgRNAs. Recognizing the importance of comprehensive off-target analysis, CHOPCHOP v2 identifies off-targets considering three mismatches (Labun et al., 2016). With the increased cutting efficiency and specificity, the CHOPCHOP v3 upgrade improves CRISPR research by addressing the issue of off-target mutagenesis. For improved data analysis, it interfaces with the UCSC browser and offers visual output for target quality comprehension. More than 200 genomes are currently supported by CHOPCHOP, which also offers gene annotations for genomic targets and three transcriptomes (human, mouse, and zebrafish) for RNA knockdown. Additionally, it expands its functionality to target RNA with Cas13 and other DNA targeting modes, making it a more versatile and powerful tool for genome editing (Labun et al., 2019). Another tool, CRISPR-ERA is for designing sgRNAs for CRISPR-based editing, repression, and activation (gene regulation studies). It employs a fast algorithm to identify sgRNA binding sites across the genome, assessing their efficiency and specificity using published data (Liu et al., 2015). Beyond its core functions, CRISPR-ERA is also suitable for plant-related CRISPR applications, genome imaging, and synthetic circuit design (Kiani et al., 2014). E-CRISP uses a rapid indexing method to identify target sequences that match the guide RNA (gRNA), ensuring efficient binding site discovery. It evaluates off-target effects and target-site similarity with the Bowtie2 alignment program, guaranteeing specific gRNA designs. Currently supporting more than 50 organisms, including plant species as well. E-CRISP can be expanded to include more species (Heigwer et al., 2014). However, the tool has limitations with a longer loading time when compared to other tools like CHOPCHOP and CRISPR-ERA.

The WGE database provides comprehensive resources for CRISPR research in mouse and human exons. It includes pre-computed off-target data and enables easy scoring and viewing of off-target sites, facilitating quick identification of high-quality CRISPR sites through filtering. WGE also features tools for generating and displaying gene targeting vectors directly in its genome browser, alongside gene models and protein translations. The system is versatile, supporting customization for any genome and is open-source and expandable (Hodgkins et al., 2015). Off-Spotter enhances gRNA design by rapidly and thoroughly identifying potential off-target sites with up to 5 mismatches. It offers extensive annotations, flexible target sequence input, and detailed transcriptomic data. Users can interactively explore different gRNA options, ensuring specificity through histograms and improving the experience with sorting and navigation features. This precision makes it highly effective for targeted genetic engineering applications (Pliatsika and Rigoutsos, 2015).

While numerous tools exist for designing sgRNAs in popular model organisms, only a few cater to non-model organisms. CRISPy-web (http://crispy.secondarymetabolites.org/) is a user-friendly tool based on CRISPy, enabling sgRNA design for any microbial genome provided by the user. With CRISPy-web, researchers can conveniently select a genomic region of interest and scan it for potential sgRNAs. The tool conducts a check for potential off-target matches and visually displays the resulting sgRNA sequences, which can be exported to text files for further analysis (Blin et al., 2016). A tool, CRISPick (https://portals.broadinstitute.org/gppx/crispick/public), ranks and selects candidate CRISPRko (CRISPR Knockout) sgRNA sequences for given targets, aiming to maximize on-target activity and minimize off-targets. It uses a preferred scoring system tailored to the enzyme and CRISPR mechanism for evaluating guides. Genome sequences from humans, mice, and rats are present (Doench et al., 2016).

### 6.1 CRISPR-Local

CRISPR-Local is a local tool designed for high-throughput single-guide RNA (sgRNA) design in plants and other organisms. It considers genetic variation and is optimized for generating genome-wide sgRNAs. The tool operates on two main principles: first, the “one-for-all” strategy constructs a comprehensive sgRNA database efficiently, generating and storing all possible sgRNAs for a given reference or user-defined genome locally; second, it retrieves or designs applicable hits by integrating data from whole genome sequencing and mRNA sequencing. CRISPR-Local offers several advantages over other sgRNA design tools, including the ability to design guide RNA for non-reference varieties, target multiple genes simultaneously, and operate offline with command-line and graphical user interface modes. It also allows for the export of multiple formats for future analysis (Sun et al., 2019).

### 6.2 Cas-Database and Cpf1-Database

Cas-Database (http://www.rgenome.net/cas-database/) is a web-based tool designed for generating optimal sgRNA sequences for Cas9 nucleases from *Streptococcus pyogenes* (SpCas9), specifically for genome-scale screening. It enables users to select multiple optimal target sequences from a vast array of genes simultaneously. The tool supports 12 different organisms and features a user-friendly interface with various filtering options (Park et al., 2016). Type V CRISPR-Cpf1 endonucleases are effective for genome editing *in vivo* across various organisms, similar to the earlier type II CRISPR-Cas9 system. However, there is a shortage of web-based tools that can efficiently select gRNAs from numerous potential genome-wide target sites. The Cpf1-Database (http://www.rgenome.net/cpf1-database/) addresses this gap by offering a tool for constructing genome-wide gRNA libraries specifically for the *Lachnospiraceae* bacterium LbCpf1 and the *Acidaminococcus* sp. bacterium AsCpf1. A simple method for creating gRNAs for AsCpf1 nucleases at the genome scale is offered by the Cpf1-sgu Database. This web interface makes it easy to retrieve the data, and the robust collection function makes it quick and simple to construct gRNAs for thousands of genes (Park and Bae, 2018). Both Cas-Database and Cpf1-database currently support sgRNA design in twelve different organisms: *Arabidopsis thaliana*, grapes, tomato, banana, and soybean, *Drosophila melanogaster*, human, rat, mouse, pig, zebrafish and *Caenorhabditis elegans* (Park and Bae, 2018).

The crisprSQL (http://www.crisprsql.com) is a new database platform designed for assessing off-target cleavage in CRISPR/Cas experiments. This platform offers insights into cutting-edge technologies driving transgenics, aids in guiding RNA design for genome engineering, and provides a transparent foundation for modelling CRISPR/Cas off-target DNA cleavage. Gene IDs attached to the data enable high-resolution analysis, informing knockout screens and functional genomics. It specifically details interactions, gene identities and epigenetic markers (Störtz and Minary, 2021). The effective targeting of sequences is crucial for any experimental success. Existing design tools often focus on specific elements, but Jackie and Albert’s Comprehensive K-mer Instances Enumerator (JACKIE) (https://github.com/albertwcheng/JACKIE2) offers a broader approach. It identifies all single- and multicopy sites in the target genome, making it suitable for large-scale genome designs. JACKIE can be integrated into genome browsers for an intuitive web-based graphical interface. It employs fast algorithms to evaluate off-target counts, allowing for the identification of designs with low off-target probabilities among millions of sequences within a practical time frame which is 100-fold more efficient than most popular tools. JACKIE offers comprehensive k-mer enumeration in the target genome and rapid evaluation of off-target effects (Zhu et al., 2022).

## 7 Experiments-based guide RNA design tools

The development of genome engineering with CRISPR technology has revolutionized our understanding of genomic functions. GuideScan, an open-source software, helps to build customized gRNA databases for any target genome, aiding in the design of both paired and single gRNA libraries. GuideScan allows users to customize target sequences for different CRISPR endonucleases by adjusting three parameters: the PAM, its position relative to a target sequence, and the gRNA length. However, due to its serial processing, GuideScan is computationally intensive for designing CRISPR gRNA libraries from large genomes (Perez et al., 2017). MultiGuideScan (https://github.com/bioinfomaticsCSU/MultiGuideScan) addresses this challenge by implementing parallel processing of GuideScan’s multiple processes. MultiGuideScan accelerates gRNA library design by 9–12 times compared to GuideScan, enabling efficient design of guide RNA libraries from large genomes (Li et al., 2020). Another tool, the dbGuide database, provides a repository of experimentally validated guide RNA sequences for CRISPR/Cas9 knockouts in humans and mice. Accessible via a user-friendly HTML interface, it utilizes data tables and JavaScript libraries to display information in both graphical and tabular formats. For more information, visit https://sgrnascorer.cancer.gov/dbguide. Notably, the database includes over 4000 guide RNA sequences validated through direct amplicon sequencing or manually from more than 1000 publications, making it a valuable resource. The framework supports ongoing updates with new, experimentally validated guide RNA sequences for CRISPR/Cas9 knockouts. It also includes sequences from various gene editing systems, different species, and other functions such as gene activation and repression, base editing, and more (Gooden et al., 2021).

The genome-wide approach in the CRISPR experiment design reduces validation time via PCR and minimizes computational overhead. A novel machine learning program has been built to address issues with existing tools in guiding repetitive or A/T-rich genomic regions. This scoring model significantly enhances prediction accuracy, even in non-crop genomes (Müller Paul et al., 2022). CROPSR (https://github.com/cabbi-bio/CROPSR) presents new techniques and workflows for conducting CRISPR/Cas9 knockout experiments, focusing on simplifying the design, assessment, and validation of gRNA sequences, especially in crop research. This standalone tool, with minimal dependencies and a modular structure, is designed for use on supercomputers. It can create extensive, searchable databases with essential genome-wide data for CRISPR experiments, including PCR validation by providing primer pairs. CROPSR outperformed other tools like CHOPCHOP md CRISPR-P for designing gRNA for each gene with a score ≥ 0.8. The score cutoff was defined according to the algorithm. The improved scoring model in CROPSR marks a significant improvement over existing methods (Müller Paul et al., 2022). Advanced CRISPR tools like the Synthego design tool streamline the process of designing guide RNAs (gRNAs). One can select from more than 120,000 genomes and 8,300 species to create gRNAs for gene knockout with minimal off-target impacts and view the positions of your sequences within the gene or validate guides designed using other tools (https://www.synthego.com/products/bioinformatics/crispr-design-tool) (Synthego design tool, 2024).

A recent study showed that CRISPRoffT is a complete online resource that brings together experimentally anticipated and confirmed off-target data from different CRISPR technologies, Cas enzyme variants, sgRNA designs, and human and mouse cell types. This platform now collects more than 226,000 guide–off-target combinations, including 8,840 experimentally validated off-targets. It is the largest such library to date. For each guide sequence and gene, CRISPRoffT lets you compare different experimental circumstances, technologies, and Cas/gRNA combinations. This gives you useful information on off-target behaviour. CRISPRoffT is a very important tool for improving gRNA design and making off-target prediction algorithms more accurate and reliable. It does this by giving precise information about the state of on-target and off-target sites (Wang et al., 2025).

## 8 Machine learning-based tools for on/off-target efficiency

CRISPOR (http://crispor.tefor.net/) is an online tool designed to aid CRISPR–Cas9 genome editing. It identifies guide RNAs in a given sequence and ranks them based on potential off-target effects and predicted on-target efficiency. The tool provides comprehensive features, including guide RNA selection, cloning, and expression, and also provides primers for evaluating guide activity and possible off-target effects. CRISPOR displays the input sequence graphically with potential guide targets and offers detailed information for each target, such as position, sequence, efficiency, and out-of-frame scores. It also integrates with the UCSC Genome Browser for interactive visualization and annotations. Recent updates include support for genome-wide CRISPR screens, custom oligonucleotide synthesis for guide cloning, and the designing of NGS primers to detect off-target mutations (Concordet and Haeussler, 2018). Another tool, DeepCRISPR is an innovative deep-learning model that simultaneously predicts CRISPR sgRNA on-target knockout efficiency and genome-wide off-target profiles. It starts with unsupervised pre-training on hundreds of millions of unlabelled sgRNA sequences to learn rich sequence representations, followed by fine-tuning a convolutional neural network with experimentally verified on- and off-target data. DeepCRISPR outperforms state-of-the-art tools consistently with varied human datasets. Its modular design makes it easily expandable to incorporate more advanced architectures, improved feature engineering, and higher-quality training data, leading to continuous enhancement as larger CRISPR screening and off-target detection data sets are obtained (Chuai et al., 2018).

A recent study created a predictive pipeline to find possible off-target sites and cleavage efficiency for CRISPR-Cpf1 nucleases, specifically AsCpf1 and LbCpf1. Cpf1’s recognizes a T-rich PAM that enhances its specificity, and therefore indicates its potential for precise genome editing. The main objective is to get more on-target activity and fewer off-targets. For that, a multilayer perceptron (MLP)-based classifier using both sequence- and base-dependent binding energy features. The training data included both types of data: experimental (positive data) and computationally predicted (negative off-target pairs). The models accurately predicted cleavage efficiency and identifies various factors, including mismatch distribution and the melting temperature of the non-seed region. It further highlights other factors such as PAM binding energy, GC content, dinucleotide frequencies, and mismatches in seed and trunk regions, which offers insights into Cpf1 off-target activity (Kesarwani et al., 2023). CRISPR-M (https://github.com/lyotvincent/CRISPR-M) is a deep learning architecture designed to improve the prediction of CRISPR-Cas9 off-target effects, particularly for target sites with mismatches and indels. It uses a unique encoding scheme and a multi-view architecture that merges convolutional neural networks and bidirectional LSTM layers into a three-branch network. The method has consistently outperformed previous methodologies in assessing datasets like CIRCLE and GUIDE_I, demonstrating robust generalization and predictive precision. The architecture’s ability to simulate the impacts of mismatch/indel sites and sequence characteristics is also validated through visual analysis. This study represents a significant advancement in sgRNA off-target prediction (Sun et al., 2024). DeepCRISTL (https://github.com/OrensteinLab/DeepCRISTL) is a deep learning model designed to predict on-target efficiency for CRISPR-Cas9 editing by utilizing high-throughput datasets (CRISPRon and DeepHF). It uses transfer learning (TL) to refine these features with functional or endogenous data pertinent to specific cellular contexts, including human, Zebrafish, and mouse. DeepCRISTL exhibits enhanced efficacy compared to current methodologies. Its architecture enables effective adaptation to smaller, context-specific datasets, contingent upon the adequate correlation between source and target data. Moreover, saliency map analyses demonstrated that the features acquired by DeepCRISTL possess biological significance (Elkayam et al., 2024). Similarly, CrnnCrispr is also a deep learning method for the prediction of CRISPR/Cas9 on-target activity. It uses four advanced deep learning models such as DeepSpCas9, TransCrispr, DeepCas9, and CRISPRont. It can help models when training data is limited (Zhu et al., 2024).

Toufikuzzaman et al. (2024) introduced the CRISPR-DIPOFF suite (https://github.com/tzpranto/CRISPR-DIPOFF), an interpretable deep learning model, that precisely predicts CRISPR-Cas9 off-target sites. It work on recurrent neural networks (RNNs) optimized through genetic algorithms, and only uses sequence data. Using Integrated Gradients to interpret the model was important because it showed two separate sub-regions within the sgRNA seed region, which gave us new information about why off-target effects happen. While the work focused solely on substitution mismatches and excluded structural or energy-based comparisons, there is the need to expand future studies to include other Cas variants (e.g., Cas12, Cas13), indel mismatches, broader genomic contexts, and diverse benchmark datasets from various species and cell types to improve generalizability and robustness.

The RNAS-sgRNA (https://github.com/shehlarafiq5/RNAS-sgRNA) model, a hybrid framework integrating neural architecture search (NAS) with recurrent neural networks (RNN), provides a reliable method for predicting the on-target efficacy of CRISPR/Cas9 sgRNAs. By automating architecture optimization via NAS, the model diminishes manual tuning while proficiently analysing sgRNA sequences represented as binary matrices. RNAS-sgRNA demonstrated superior performance across various datasets and cell lines, when compared area under the receiver operating characteristic curve (AUROC), with an average AUROC enhancement of 14.74% compared to DeepCRISPR. The model exhibited robust performance on smaller datasets via transfer learning, highlighting its potential applicability in personalized medicine and genome-wide contexts where data is frequently scarce (Rafiq and Assad, 2025).

## 9 Some other recent tools

DrugThatGene (DTG) (https://github.com/pinellolab/DrugThatGene) is an online tool designed to help translate functional genomics findings for potential treatments. It helps in the analysis of therapeutic targets identified using functional genetic screens. By submitting a list of genes, users can use DTG to automatically identify small molecules and access supporting information from various databases. DTG also aids in recognizing common biological pathways and protein complexes, thus speeding up the identification of small molecules from extensive CRISPR screen data (Canver et al., 2019). Additionally, “WeReview: CRISPR Tools” is an online platform offering a comprehensive, up-to-date repository of computational tools for designing CRISPR/Cas experiments. Researchers can search for tools that meet their specific needs and suggest modifications or new tools through the website (Torres-Perez et al., 2019).

CRISPR-Cas systems enable allele-specific gene editing, offering a personalized treatment approach for autosomal dominant disorders by targeting and correcting disease-causing alleles. AsCRISPR (https://bio.tools/AsCRISPR) facilitates the design of sgRNAs for allele-specific genome engineering, taking into account factors like allele discrimination, efficiency, and off-target effects, providing a comprehensive and user-friendly platform (Zhao et al., 2020). Recent prime editing (PE) technology uses prime editing guide RNA (pegRNA) to direct a fusion protein, along with nCas9 and reverse transcriptase, to specific genomic sites for precise editing. Designing PEs is more complex than using single gRNAs with CRISPR nucleases or base editors, and analysing high-throughput sequencing data post-PE requires special consideration of PEs’ unique features. To solve these complexities, two user-friendly web tools, PEDesigner and PE-Analyzer, have been created. PEDesigner helps select pegRNA by providing extension sequences, target sequences, and nicking gRNA sequences. PE-Analyzer evaluates PE results, summarizing data related to mutations in tables and interactive graphs (Hwang et al., 2021). SpacePHARER (CRISPR Spacer Phage–Host Pair Finder) (https://github.com/soedinglab/spacepharer) is a fast and sensitive tool for predicting relationships between phage and host by identifying phage genomes matching CRISPR spacers in different data (genomic or metagenomic). It can compare phages at the protein level, adjust its scoring system for very short sequences, and combine evidence from multiple matches to reduce false positives. Run time for SpacePHARER is 12 min to process the dataset which is 47 times faster than BLASTN search (575 min) (Zhang et al., 2021). SynBioStrainFinder (http://design.rxnfinder.org/biosynstrain/) is the latest tool developed to integrate CRISPR/Cas gene-editing system information with genome sequences and data to form a comprehensive database of microbial strains. SynBioStrainFinder is a publicly available resource that offers an easy-to-use interface for searching, exploring, and visualizing comprehensive data on microbial strains at http://design.rxnfinder.org/biosynstrain/. The quick strain information query system integrates modules to create a curated and accessible platform. It has retrieved 1426 records of CRISPR/Cas-based gene editing from 157 microbial strains. The database also includes 773,298 strain-related compounds and 139,499 genome sequences (Cai et al., 2022).

## 10 Conclusion

Today, the CRISPR tools are diverse, functional, highly specialized, with difficulty in scalability, and ease of use. These resources provide researchers with valuable support in designing, executing, and analysing genome editing experiments, enabling more efficient and precise modifications of the genetic code. From comprehensive databases of validated guide RNA sequences to user-friendly web-based tools for primer design and outcome analysis, the landscape of genome editing resources continues to expand and evolve. CRISPOR, CHOPCHOP and DeepCRISPR offer robust features for gRNA design and are excellent choices for many users. For users focused on ease of use and visual tools, CHOPCHOP is a great option, while CRISPOR is highly reliable for detailed off-target analysis and supports a wide range of organisms. Ultimately, the best tool depends on specific requirements and workflow preferences. Many tools are limited by specific species (primarily human or plant), by dated datasets, or by a focus on specific Cas proteins such as SpCas9 but not on emerging variants such as Cas12a, Cas13, or anti-CRISPR. Off-target prediction remains the biggest challenge, with limited validation. Various tools are developed based on machine learning algorithms, often restricted to a fewer Cas variant and limiting their practical applications. There are functional annotation gaps in CRISPR technology present in terms of limited understanding of the roles, mechanisms, and biological functions of newly discovered CRISPR-Cas systems, particularly those identified through metagenomic and bioinformatic approaches. These gaps are particularly evident in a typical system, where little experimental data exists to confirm computational predictions. Because of the uncertainties surrounding their activity, PAM preferences, and off-target behaviour, many CRISPR variants are therefore unreliable for use in genome editing applications. Current CRISPR technology has limitations due to its lack of integration with experimental validation, affecting the reliability and translational potential of computational predictions. In silico tools can predict guide RNA efficiency, PAM recognition, and off-target effects, but they are rarely linked with standardized or high-throughput experimental workflows. This gap between functional verification and computational design causes uncertainty in editing results. Therefore, integrated platforms that combine automated experimental validation pipelines are needed. For the future CRISPR tools, there should be a focus on unifying platforms that handle multiple types of Cas, greater organism coverage, and design facilitated by artificial intelligence for improved guide RNA specificity and reduction of off-targets. Also, combining computational tools with experimental methods provides the most comprehensive understanding of CRISPR experiments. These advancements not only accelerate research progress but also hold promise for various applications in biotechnology, medicine, greater scalability for throughput experiments, and inclusion of real-time CRISPR screening results will further close the gap between research, synthetic biology, and therapy. As the field of genome editing continues to grow, the continued development and refinement of these databases and tools will be crucial for unlocking the full potential of genetic engineering and realizing its benefits for society.
